# Incarceration history and opioid use among adults living with HIV and chronic pain: a secondary analysis of a prospective cohort study

**DOI:** 10.1186/s40352-024-00272-x

**Published:** 2024-05-29

**Authors:** Anna B. Lichtiger, Yuting Deng, Chenshu Zhang, Justina Groeger, Hector R. Perez, Gayatri Nangia, Melanie Prinz, Emma Richard, Matthew Glenn, Ana Alicia De La Cruz, Ariana Pazmino, Chinazo O. Cunningham, K Rivet Amico, Aaron Fox, Joanna L. Starrels

**Affiliations:** 1 Division of General Internal Medicine, Albert Einstein College of Medicine & Montefiore Medical Center, 3300 Kossuth Ave, Bronx, NY 10467 USA; 2 Stony Brook School of Health Professions, Stony Brook, NY USA; 3 Carelon Research, Wilmington, DE USA; 4 Department of Physical Medicine and Rehabilitation, NYU Grossman School of Medicine, New York, NY USA; 5 New York State Office For People With Developmental Disabilities, Bronx, NY USA; 6 Division of Infectious Diseases, Columbia University Irving Medical Center, New York, NY USA; 7 New York State Office of Addiction Services and Supports, Albany, NY USA; 8 Department of Health Behavior and Health Education, School of Public Health, University of Michigan, Ann Arbor, Michigan USA

**Keywords:** Incarceration, Opioids, Chronic pain, HIV

## Abstract

**Background:**

Adults living with HIV have disproportionately high chronic pain, prescription opioid use, history of substance use, and incarceration. While incarceration can have long-lasting health impacts, prior studies have not examined whether distant (>1 year prior) incarceration is associated with opioid use for chronic pain, or with opioid misuse or opioid use disorder among people living with HIV and chronic pain.

**Methods:**

We conducted a secondary analysis of a prospective cohort study of adults living with HIV and chronic pain. The independent variables were any distant incarceration and drug-related distant incarceration (both dichotomous). Dependent variables were current long-term opioid therapy, current opioid misuse, and current opioid use disorder. A series of multivariate logistic regression models were conducted, adjusting for covariates.

**Results:**

In a cohort of 148 participants, neither distant incarceration nor drug-related incarceration history were associated with current long-term opioid therapy. Distant incarceration was associated with current opioid misuse (AOR 3.28; 95% CI: 1.41-7.61) and current opioid use disorder (AOR 4.40; 95% CI: 1.54-12.56). Drug-related incarceration history was also associated with current opioid misuse (AOR 4.31; 95% CI: 1.53-12.17) and current opioid use disorder (AOR 7.28; 95% CI: 2.06-25.71).

**Conclusions:**

The positive associations of distant incarceration with current opioid misuse and current opioid use disorder could indicate a persistent relationship between incarceration and substance use in people living with HIV and chronic pain. Additional research on opioid use among formerly incarcerated individuals in chronic pain treatment is needed.

## Background

People living with HIV (PLWH) have a high prevalence of chronic pain. In several cohorts of PLWH, 25-90% live with chronic pain (Dahlhamer et al. 2016; Jiao et al. 2016; Addis et al. 2020). This may be explained by direct effects of HIV on the nervous system, opportunistic infections, HIV therapies, and age-related pain conditions such as arthritis (Tsao et al. 2010). Consequentially, the prevalence of prescription opioid use is also high among PLWH. According to a study of Medicaid patients, PLWH received 50-65% more opioid prescriptions and were more likely to receive long-term opioid therapy (LTOT) than HIV-negative matched controls (Canan et al. 2019).

Incarceration history is more common among PLWH than in the general population. Each year, approximately one in seven PLWH are released from a correctional or detention facility (Spaulding et al. 2009). The prevalence of HIV among prisoners is 3 times higher than in the general population (Maruschak 2023; Westergaard et al. 2013). PLWH may be overrepresented among incarcerated populations in part because substance use, especially intravenous drug use, is a shared risk factor for both HIV and incarceration (Cofrancesco et al. 2008; Bing et al. 2001; Bositis and St Louis 2019)﻿. In the US, the War on Drugs criminalized drug-related activities including possession, manufacturing, and distribution of certain drugs. The rate of incarceration for drug-related crimes in the US increased nearly tenfold from 15 to 143 per 100,000 people between 1980 and 2010 (Council 2014).

Despite the high prevalence of chronic pain, substance use, and incarceration among PLWH, the association between incarceration history and subsequent LTOT, opioid misuse, or opioid use disorder (OUD) has not been examined among PLWH and chronic pain. There are reasons to expect that remote incarceration could be associated with decreased use of LTOT for PLWH who have chronic pain, compared to people without history of incarceration. It is known that providers are cautious in prescribing opioids to patients that they consider high risk, and there is evidence that other sociodemographic factors, such as race and gender, are associated with LTOT prescribing and deprescribing, even more so than clinical factors such as concurrent use of benzodiazepines. (Buonora et al. 2019; Hastings et al. 2020). We hypothesize that remote incarceration, particularly drug-related incarceration, will be similarly negatively associated with LTOT. It is also possible that remote incarceration could be associated with greater LTOT. An analysis of the 2015-2016 National Survey on Drug Use and Health (NSDUH) found that among the general public, criminal justice involvement (self-reported arrest, incarceration, probation, or parole) in the past year was positively associated with past year self-reported prescription opioid use (inclusive of prescribed or un-prescribed, and long-term or short-term use) (Winkelman et al. 2018). However, prior studies have not examined the long-term association of incarceration and prescription opioid use among people with chronic pain.

Prior studies of the general population have also found incarceration history to be associated with opioid misuse and OUD (Winkelman et al. 2018; Hastings et al. 2020). These findings could be explained at least in part by criminal justice involvement due to substance use, though co-occurring factors that contribute to both opioid misuse and OUD and incarceration include structural determinants such as poverty, racism, and inadequate health care for medical and mental health conditions (Winkelman et al. 2018; Joudrey et al. 2019). However, studies have not examined whether a distant incarceration (> 1 year prior) remains associated with opioid misuse or OUD in PLWH and chronic pain.

In this paper, we aim to examine the associations of distant incarceration history with current LTOT, current opioid misuse, and current OUD among PLWH and chronic pain. We hypothesize that formerly incarcerated PLWH would be less likely to be prescribed current LTOT for chronic pain than those without incarceration history. We further hypothesize that distant incarceration history would be associated with current opioid misuse and current OUD.

## Methods

### Overview and study design

We conducted a secondary analysis of data from the Pain and Medication Effects on Treatment Outcomes (PIMENTO) study, a 12-month observational prospective cohort study. The Albert Einstein College of Medicine Office of Human Research Affairs, the institutional review board, approved the study. Current prisoners were not recruited for this study.

### Setting

The PIMENTO study was conducted at Montefiore Medical Center in the Bronx, New York. Montefiore is the largest academic hospital system serving the Bronx community, which has experienced high rates of HIV and incarceration. In 2020, Bronx County had an HIV prevalence of 2,114 per 100,000 people versus 151 per 100,000 in New York City (New York City Department of Health and Mental Hygiene 2022). In 2020, 268 per 100,000 people in the Bronx were in prison versus 193 per 100,000 in New York State (Widra and Encalada-Malinowski 2022).

Study participants received clinical care in Montefiore specialty HIV clinics or primary care practices. Potential study participants were identified using the Montefiore electronic health record (EHR) and recruited via telephone, followed by in-person discussion and written informed consent prior to data collection. In the recruitment process, no participants were asked about prior incarceration history and no prisoners were enrolled in the study.

### Study participants

PIMENTO study inclusion criteria were: 1) 18 years or older; 2) HIV infection; 3) chronic pain, defined by International Classification of Diseases, tenth revision (ICD-10) codes for back pain, osteoarthritis, or neuropathic pain and self-reported pain for at least 3 months; 4) receiving outpatient HIV care at Montefiore Medical Center, defined by at least 2 visits in the past 12 months; and 5) English-speaking. To enhance enrollment of participants prescribed opioids for the primary analysis, after 120 participants were enrolled, an additional inclusion criterion was added to require an opioid prescription in the prior 30 days (EHR data). Exclusion criteria were: 1) malignancy by EHR or self-report, and 2) inability to provide informed consent. For the current analysis, we excluded one individual due to incarceration history in the past month, because the focus of the study is on distant incarceration.

### Data collection

Participants completed five study visits, 3 months apart, over a 12-month period. At each study visit, data collection included Audio Computer-Assisted Self-Interviewing (ACASI) and the interviewer-administered Psychiatric Research Interview for Substance and Mental Disorders (PRISM-5-OP) (Hasin et al. 2020).

ACASI interview domains included: sociodemographic variables, worst pain location, depressive symptoms (Center for Epidemiologic Studies Depression Scale [CES-D] (0-30), high symptoms ≥ 16) (Zhang et al. 2012), anxiety symptoms (Brief Symptom Inventory- Anxiety Subscale [BSI-Anxiety] (0-24), continuous) (Derogatis and Melisaratos 1983), post-traumatic stress disorder symptoms (PTSD Checklist-Civilian [PCL-C] (0-24), high symptoms ≥ 14) (Lang and Stein 2005), pain characteristics, pain severity and function (average pain intensity (P), interference with enjoyment of life (E), and interference with general activity (G) [PEG] score) (Krebs et al. 2009), physical function and disability due to chronic pain (Roland-Morris Disability Scale) (Stroud et al. 2004), pain catastrophizing (Pain Catastrophizing Scale [PCS]) (Sullivan et al. 1995), current opioid misuse (Current Opioid Misuse Measure [COMM]) (Butler et al. 2007), as well as internalized HIV stigma (Internalized AIDS-Related Stigma Scale [IA-RSS]) (Kalichman et al. 2009).

Current OUD and other substance use disorders were assessed using the PRISM-5-OP, a clinical diagnostic interview for DSM-5 that assesses prescription OUD, heroin, and cocaine use disorders and is relevant for patients with chronic pain prescribed opioids. The PRISM-5-OP also assessed sociodemographic characteristics including age, gender, race, homelessness, marital status, educational status, and incarceration history and reasons for incarceration*.*

Montefiore Medical Center EHR data were extracted to assess for HIV viral load, written and dispensed opioid prescriptions, and medical comorbidities (ICD-10 codes to determine Charlson comorbidity score based on 17 comorbidities associated with ten-year mortality) (Charlson et al. 1987). Viral load data were extracted from 45 days prior to enrollment through enrollment. Opioid prescriptions and medical comorbidities data were extracted in the 12 months prior to enrollment through 90 days after enrollment. The opioid dose in Morphine Milligram Equivalents (MME) for dispended prescription opioids was ascertained from prescription monitoring program (PMP) data.

### Measures

#### Independent variables

The primary independent variable was distant incarceration (yes/no), in response to the question: “Approximately how much time in total were you incarcerated in your life?” If participants responded with a numerical value greater than 0 and reported incarceration > 1 year prior to enrollment, they were considered to have distant incarceration history. Second, we classified a participant as having a history of drug-related incarceration (yes/no) based on qualitative review of their verbatim response to the open-ended question, “Why were you in jail or prison?” An incarceration was classified as drug-related if the reported reason mentioned controlled substances or drugs, with or without specific mention of possession, buying, selling or intent to sell. Incarceration history was assessed at the baseline research visit. Two participants who reported distant incarceration did not report the reason for incarceration and we conservatively considered those not to be drug-related.

#### Dependent variables

Three dichotomous outcome variables were assessed at each of the five quarterly study visits (at 0, 3, 6, 9, and 12 months) and considered positive for this analysis if they were positive at any of the five study visits. The primary outcome was current LTOT, defined as receiving at least 3 prescriptions for opioid analgesics, each separated by at least 21 days, in the 6-month period preceding a study visit (yes/no) per EHR or PMP. Secondary outcomes were current opioid misuse and current OUD. Current opioid misuse was assessed using three items from the Current Opioid Misuse Measure, consistent with an approach by Frimerman et al (Frimerman et al. 2021). The questions asked how often, in the past 30 days, have you: 1) had to take more of your opioid pain medication than prescribed, 2) borrowed opioid pain medication from someone else, and 3) used your opioid pain medicine for symptoms other than pain (e.g., to help you sleep, improve your mood, or relieve stress) (Frimerman et al. 2021)? Response options greater than “never” for any of the three items were considered positive for current opioid misuse (Frimerman et al. 2021). There is no gold standard for measuring opioid misuse; this three-item scale was selected because these items are specific for misuse behaviors (Frimerman et al. 2021). Frimerman et al found the internal reliability of this subset of the COMM to be acceptable (Frimerman et al. 2021). Current OUD was considered positive if the DSM-5 score was 2 or more, consistent with at least mild OUD, for prescription OUD, heroin use disorder, or both (Hasin et al. 2020).

#### Covariates

We considered the following covariates: demographic characteristics (age, gender [male vs. female, including transgender female]) and health characteristics (high PTSD symptoms, PEG score, Charlson Comorbidity Index, and current LTOT, which was considered a covariate for the variables current opioid misuse and current OUD).

### Statistical analysis

To describe differences in participant characteristics and current LTOT, current opioid misuse, and current OUD among participants with and without distant incarceration history, we first conducted bivariate analyses using chi-square tests or t-tests as appropriate. Next a series of multivariate logistic regression models were run with covariates and the incarceration variables as independent variables and the opioid-related measures (current LTOT, current opioid misuse, and current OUD) as dependent variables. The reference group in each model was no distant incarceration. To determine which covariates to include in the multivariate models, we performed bivariate analyses with incarceration history and current LTOT as independent variables and demographic and health variables as dependent variables. None of the demographic and health variables were found to be significantly associated and so were not classified statistically as confounders in our sample. However, because demographic factors, comorbidity burden, experience of pain and how it interferes with daily life, and substance use have been shown to influence current LTOT, current opioid misuse, and current OUD, we considered these as confounders and therefore included age, gender, the Charlson Comorbidity Index, PEG score, and current LTOT as covariates in the models (Hastings et al. 2020; López-Martínez et al. 2019; Karmali et al. 2020; Han et al. 2018).

## Results

Of 148 study participants, 57% identified as female, 54% as Black/African American, 33% as Hispanic/Latinx, and 7% as white (Table 1). The mean age was 56 (SD 8.4). Most (80%) had HIV viral suppression at baseline. Sixty one percent of participants (*n*=91) were disabled and not working. The most common location for worst chronic pain was low back (44% of participants), followed by leg (20%). The average PEG score was 7.3 out of 10 (SD 1.6), indicating severe pain and pain impact. Forty-three percent had high PTSD symptoms.

**Table 1:** Participant characteristics by incarceration history

	**Total (*N*=148)** **n (%)**	**Distant Incarceration (*n*=51)** **n (%)**	**No Incarceration (*n*=97)** **n (%)**
**Male gender**	63 (43)	29 (57) *	34 (35)
**Age, mean (SD)**	56 (8.4)	57 (9.2)	55 (7.9)
**Race/ethnicity**			
Black/ African American	80 (54)	26 (51)	54 (56)
Hispanic/ Latinx	49 (33)	18 (35)	31 (32)
White/Caucasian	10 (7)	3 (6)	7 (7)
Other	6 (4)	3 (6)	3 (3)
**Born outside mainland US**	115 (78)	41 (80)	74 (76)
**Completed high school or equivalent**	56 (38)	14 (27)	42 (43)
**Married or living with partner**	38 (26)	12 (24)	26 (27)
**Experiencing homelessness**	10 (7)	4 (8)	6 (6)
**HIV viral load undetectable**	118 (80)	38 (75)	80 (82)
**HIV stigma (Internalized AIDS-Related Stigma Scale) (0-6), mean (SD)**	3.5 (2.0)	3.4 (1.9)	3.5 (2.0)
**Comorbidities (Charlson Comorbidity Index 0-37), mean (SD)**	8 (2.1)	7.7 (1.8)	8.2 (2.3)
**Worst pain location (%)**			
Back, neck, or shoulder	87 (59)	32 (63)	55 (57)
Lower extremity (legs or hip)	44 (30)	14 (27)	30 (31)
Other	14 (9)	4 (8)	10 (10)
**PEG (0-10), mean (SD)**	7.3 (1.6)	7.2 (1.5)	7.3 (1.7)
**Pain catastrophizing (Pain Catastrophizing Scale, 0-52), mean (SD)**	27 (18)	28 (13)	27 (13)
**Physical function and disability (Roland-Morris Disability Scale, 0-11), mean (SD)**	9.1 (2.1)	9.2 (2.1)	9.0 (2.1)
**Cocaine use disorder (current)**			
Mild	5 (3)	4 (8) *	1 (1)
Moderate	5 (3)	4 (8) *	1 (1)
Severe	3 (2)	1 (2)	2 (2)
**Opioid use disorder (current)**			
Mild	27 (18)	15 (29) **	12 (12)
Moderate	10 (7)	4 (8)	6 (6)
Severe	8 (5)	3 (6)	5 (5)
**High PTSD symptoms (PTSD Checklist-Civilian ≥ 14)**	63 (43)	16 (31) *	47 (48)
**High depressive symptoms (Center for Epidemiologic Studies Depression ≥ 16)**	36 (24)	7 (14) *	29 (30)
**Anxiety symptoms (Brief Symptom Inventory –Anxiety, 0-24), mean (SD)**	5.0 (5.6)	4.2 (5.5)	5.5 (5.6)

*Abbreviation*: *PEG* Pain, Enjoyment, and General Activity

^*^p≤0.05 for comparison of distant incarceration vs. no incarceration

**p≤0.01 for comparison of distant incarceration vs. no incarceration

Fifty-one participants (34%) had distant incarceration and 27 (18% of the total, and 53% of those with distant incarceration) reported drug-related incarceration. Among those with distant incarceration, the mean duration of incarceration was 6.7 years (SD 6.4 years), ranging from one day to 25 years. Most participants (65%) reported being last incarcerated more than 10 years ago and the remaining 35% reported being last incarcerated between one and 10 years ago. Compared to participants with no incarceration, those with distant incarceration were more likely to be male (57% vs. 35% *p*=0.01), less likely to have high depressive symptoms (14% vs. 30% *p*=0.02) and less likely to have high PTSD symptoms (31% vs. 48% *p*=0.04), more likely to have baseline mild cocaine use disorder (8% vs. 1% *p*=0.03), baseline moderate cocaine use disorder (8% vs. 1% *p*=0.03), and baseline mild OUD (29 vs. 12% *p*<0.01) (Table 1).

**Fig. 1:**
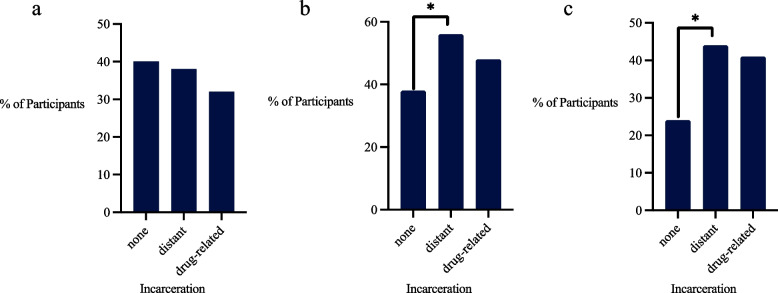
Prevelance of opioid outcomes. Abbreviations: LTOT long-term opioid therapy, OUD opioid use disorder. *p<0.05 (there was a significant difference in misuse as well as OUD among those with distant and no incarceration) a: Current long-term opioid therapy b: Current opioid misuse c: Current OUD

Among the full sample at baseline, 58 (39%) were prescribed current LTOT and the mean MME daily dose for those prescribed current LTOT was 55 (SD 82). Sixty-four (44%) had current opioid misuse and 36 (24%) had current OUD. Of the 17 participants at baseline with moderate or severe OUD, 6 (35%) were on medication assisted therapy (buprenorphine or methadone). Of the 58 participants with current LTOT, 19 (33%) had distant incarceration, and 7 (12%) had drug-related incarceration (Fig. 1a-c).

In multivariate models, neither distant incarceration nor drug-related incarceration was associated with current LTOT during the study timeframe. Distant incarceration was associated with current opioid misuse (AOR 3.28; 95% CI: 1.41-7.61, *p*<0.01) and current OUD (AOR 4.40; 95% CI: 1.54-12.56, *p*<0.01). History of drug-related incarceration was also associated with current opioid misuse (AOR 4.31; 95% CI: 1.53-12.17, *p*<0.01) and current OUD (AOR 7.28; 95% CI: 2.06-25.71, *p*<0.01) (Table 2).

**Table 2:** Opioid outcomes by incarceration history, results of multivariate models

**Incarceration History**	**Current LTOT**	**Current Opioid Misuse**	**Current OUD**
	**AOR (95% CI)**	**AOR (95% CI)**	**AOR (95% CI)**
**Distant incarceration**	0.79 (0.37, 1.69)	3.28 (1.41, 7.61) **	4.40 (1.54, 12.56) **
**Drug-related incarceration**	0.48 (0.18, 1.32)	4.31 (1.53, 12.17) **	7.28 (2.06, 25.71) **

*Abbreviations*: *LTOT* Long-term opioid therapy, *OUD* Opioid use disorder

^*^p<0.05, **p≤0.01

Models are adjusted for age, gender, high PTSD symptoms, PEG, and Adjusted for age, gender, high PTSD symptoms, PEG, and Charlson Comorbidity Index. Current opioid misuse and current OUD are adjusted for current LTOT

## Discussion

In this cohort of PLWH and chronic pain, about a third of participants had experienced distant incarceration, and more than half of them reported incarceration that was drug-related. Contrary to our hypothesis, distant incarceration was not significantly associated with receipt of current LTOT in the first study, to our knowledge, to examine this association. Additional research is needed to understand whether and how providers’ and patients’ decisions about opioid prescribing for chronic pain are influenced by incarceration history. Importantly, this study is also first to report that even distant incarceration is associated with current opioid misuse and current OUD in PLWH and chronic pain, in a cohort of primarily middle-aged and older adults.

This study examined the relationship between distant incarceration history with current LTOT in PLWH and chronic pain. We are not aware of any prior studies of this relationship especially in the context of PLWH or chronic pain. Our finding that distant incarceration was not associated with current LTOT stands in contrast to the 2015-2016 NSDUH study which found that past-year criminal justice was associated with 1.4 times the odds of any prescription opioid use (inclusive of prescribed or un-prescribed, and long-term or short-term use) (Winkelman et al. 2018). There are many possible explanations for the discrepancy between our finding and the 2015-2016 NSDUH study. Recent, but not remote, incarceration could be associated with short-term prescribed opioids or non-prescribed use, e.g., if individuals have pain related to injury during incarceration or pain related to the reason for incarceration. It is also possible that the association seen in the general population may not hold true among people with chronic pain in general, or among PLWH who have chronic pain. In the context of chronic pain, providers may not consider or even be aware of remote incarceration, and it may not impact LTOT at all. It is possible that providers treating PLWH may be particularly unlikely to consider remote incarceration in decisions about opioid prescribing, if their focus on retaining patients in care to control their HIV overrides concerns about risks of prescribing decisions (Starrels et al. 2016). Finally, it is possible that this secondary analysis was underpowered to detect a relationship between incarceration and LTOT. The fact that our findings did not support the hypothesis that distant incarceration history would be associated with LTOT is somewhat reassuring, as it may suggest that providers are not making prescribing decisions based on patients’ incarceration history.

We found that distant and drug-related incarceration were associated with a three to four-fold increase in odds of current opioid misuse and with a three to seven-fold increase in odds of current OUD. The findings that incarceration history was associated with current opioid misuse and current OUD is consistent with prior studies (Winkelman et al. 2018; Hastings et al. 2020). The 2015-2016 NSDUH study reported past year criminal justice involvement was associated with 1.6 times the odds of opioid misuse, 2.2 times the odds of OUD, and 4.2 times the odds of heroin use in the past year (Winkelman et al. 2018). A retrospective study of over 80,000 Medicaid recipients in Rhode Island who received an opioid prescription found that past-year release from prison and arrest were associated with approximately doubling of the odds of OUD or overdose (Hastings et al. 2020). PLWH also more likely have opioid misuse and OUD (Canan et al. 2019; Aldosari et al. 2024). Our study extends these prior findings by reporting that not only past-year incarceration, but also distant incarceration, is associated with current opioid misuse and current OUD, and this association is present even in the context of chronic pain treatment for PLWH. These findings may indicate that the associations between distant incarceration and current opioid misuse and current OUD may be more persistent than previously thought.

Our study methods cannot directly establish a causal relationship between prior incarceration and current opioid misuse or current OUD. Prior incarceration in this dataset may simply reflect a history of substance use that led to incarceration and confers risk of current opioid misuse and current OUD. Other confounders may be present including poorly treated chronic pain, which is plausible as recently incarcerated individuals may be less connected to outpatient healthcare and have greater mistrust in medical systems (Wildeman and Wang 2017; Jaiswal 2019).

However, it is plausible that even distant incarceration can have long-term impact on current opioid misuse and current OUD. Incarceration can be thought of as a toxic exposure, similar to the concept of stress as a toxin that can alter physiology and drive disease (Wright 2011). Incarceration history has been associated with many health conditions, including left ventricular hypertrophy, infectious diseases such as hepatitis (Wildeman and Wang 2017), mental health conditions such as major depressive disorder (Wildeman and Wang 2017; Turney et al. 2012), as well as pain, and functional impairment (Williams et al. 2014). Current opioid misuse and current OUD could be driven in part by exposure to incarceration history and could be compounded by these aforementioned physical and mental health conditions. The experience of incarceration and the collateral consequences, such as discrimination in housing and employment and weakening of social ties, can persist long after incarceration and could influence risk of current opioid misuse or current OUD (Howell et al. 2022). Further, drug use is criminalized and therefore a risk factor for not only incarceration, but future drug use as well. There may be other factors mediating the prevalence of misuse and OUD among our cohort of PLWH and chronic pain, such as poverty, racism, mental health diagnoses, lack of insurance, and living in communities where drug use is more common (Winkelman et al. 2018; Joudrey et al. 2019; Cernasev et al. 2020).

When clinicians encounter patients with incarceration history, it should prompt exploration of certain associated conditions, such as SUD. Identifying patients with OUD is important so that they can be offered appropriate medication treatment with methadone or buprenorphine. And in the context of chronic pain treatment, learning about prior incarceration in addition to prior treatments, and psychosocial supports may add to the full context of their chronic pain history and is likely to improve pain care (Puglisi et al. 2017; Howell et al. 2022).

This study has several limitations. First, this is a secondary analysis. The parent study was designed to examine how opioid use is associated with HIV outcomes and was not powered to examine the relationships between incarceration history and opioid use outcomes. Larger studies may be needed to primarily examine the relationships between incarceration history on current LTOT for chronic pain. Second, we recruited participants from outpatient clinics in a single hospital system in New York City and all patients had HIV and were English-speaking. The findings may not be generalizable to other settings or populations. Third, incarceration data were self-reported and there may have been misclassification if participants did not have fully disclose incarcerations or reasons for incarceration due to stigma, social desirability, and recall bias. Fourth, not all participants disclosed reasons for incarceration. Among those who did disclose, responses were often brief and did not always capture if drug-related incarcerations were due to drug possession or drug selling. However, the categories of drug use and selling are to an extent interchangeable, as many who use drug also sell drugs (Kolla and Strike 2020). Finally, it is unclear whether providers would be aware of participants’ distant incarceration history, limiting conclusions that can be drawn about provider decision-making.

## Conclusions

This study provides the first examination of the association between distant incarceration and receipt of current LTOT. Our findings suggest that among people living with chronic pain and HIV, distant incarceration history may not be related to current LTOT. Our study is also the first to demonstrate that even distant incarceration is associated with current opioid misuse and current OUD among PLWH and chronic pain to our knowledge. Future studies are needed to address the questions of how healthcare providers treat chronic pain among people with incarceration history and drug-related incarceration in particular, and how opioids are used among formerly incarcerated patients.

## Data Availability

The datasets used and/or analyzed during the current study are available from the corresponding author on reasonable request.
